# Supplementary material for: The 79°N Glacier cavity modulates subglacial iron export to the NE Greenland Shelf
Source: Nat Commun. 2021 May 24;12:3030. doi: 10.1038/s41467-021-23093-0 (PMC8144390; doi:10.1038/s41467-021-23093-0)
Supplement: Supplementary file 1 — Supplementary Information [file 41467_2021_23093_MOESM1_ESM.pdf]

Supplementary Information

to

The 79°N Glacier cavity modulates subglacial iron export to the NE Greenland Shelf

Stephan Krisch[1], Mark James Hopwood[1], Janin Schaffer[2], Ali Al-Hashem[1], Juan Höfer[3], Michiel M. Rutgers van der Loeff[2], Tim M. Conway[4], Brent A. Summers[4], Pablo Lodeiro[1,5], Indah Ardiningsih[6], Tim Steffens[1], Eric Pieter Achterberg[1*]

[1]GEOMAR Helmholtz Centre for Ocean Research Kiel, 24148 Kiel, Germany.
[2]Alfred-Wegener-Institute, Helmholtz Centre for Polar and Marine Research, 27570 Bremerhaven, Germany.
[3]Escuela de Ciencias del Mar, Pontificia Universidad Católica de Valparaíso, Valparaíso, Chile.
[4]College of Marine Science, University of South Florida, St Petersburg, FL 33701, USA.
[5]Department of Chemistry, University of Lleida – Agrotecnio-Cerca Centre, 25198, Lleida, Spain.
[6]NIOZ Royal Netherlands Institute for Sea Research, Utrecht University, 1790 AB Den Burg, Texel, The Netherlands.

[*]*Correspondence to*: Eric Achterberg (eachterberg@geomar.de)

Methods

Quality assurance

Validation of method accuracy for seawater analyses was achieved through the reference material 7602a (National Metrology Institute of Japan) for macronutrients (given in the datasheet), and SAFe S for soluble, dissolved and total dissolvable trace metals (Supplementary Table 1)[1]. Method sensitivity for seawater trace metals was monitored through SeaFAST-ICP-MS procedural blanks (Supplementary Table 2) following ref.[2]. Labile particulate analyses were validated against BCR-414 reference and indicative values[3], and monitored against process blanks (filter digest).

Supplementary Table 1: Analyzed reference materials SAFe S (entire PS100/GN05 dataset), and BCR-414 (stations S1-S6, S10) for Fe analyses, n = number of measurements.

**Reference Material**	**Consensus value**	**Reported value**
SAFe S (#273)	0.095 ± 0.008 nM [A]	0.101 ± 0.016 nM (n = 10)
BCR-414	1.85 ± 0.19 $mg \cdot g^{-1}$	2.06 ± 0.10 $mg \cdot g^{-1}$ (n = 6)

[A] Conversion from $nmol \cdot kg^{-1}$ using density of 1.026 $kg \cdot L^{-1}$

Supplementary Table 2: Method sensitivity determined as procedural blank for soluble, dissolved and total dissolvable Fe analyses (entire PS100/GN05 dataset), and digestion process blank for particulate Fe fractions (stations S1-S6, S10), n = number of measurements.

SeaFAST-ICP-MS System blank	64 ± 20 pM (n = 465)
Digestion Process Blank	23 ± 3 pM (n = 7)

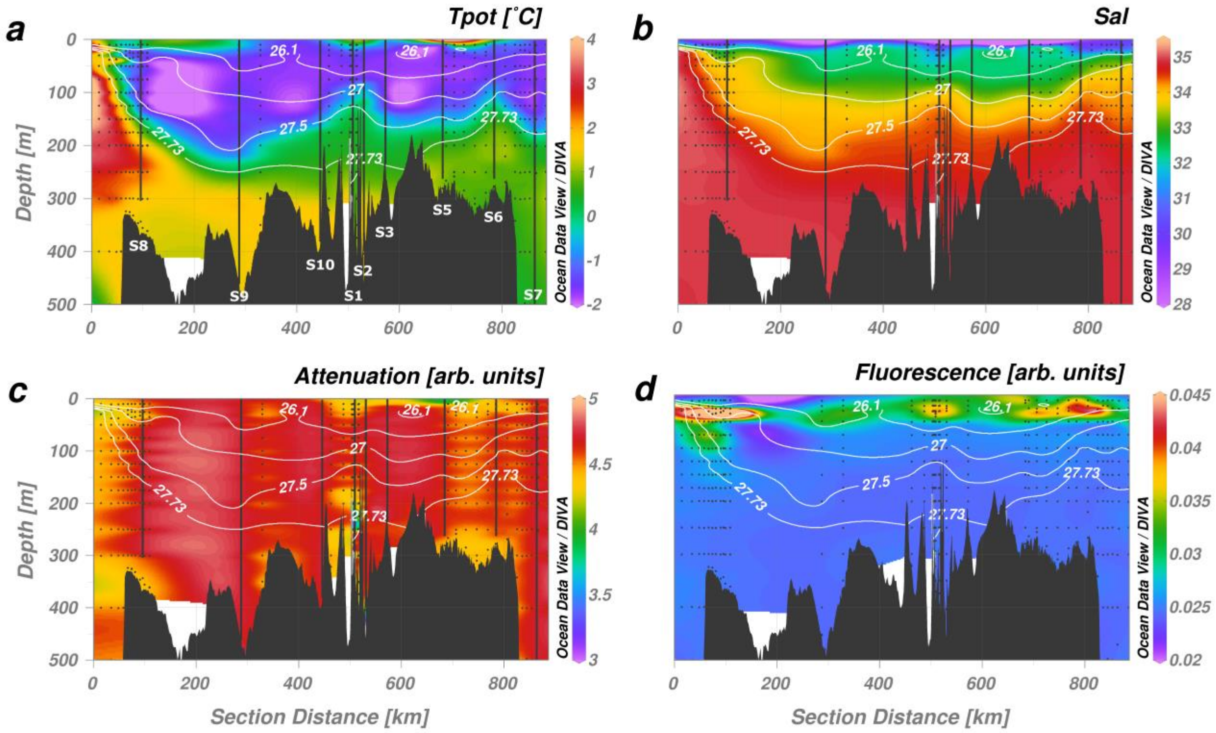

Supplementary Figure 1: NE Greenland Shelf section of (a) potential temperature, (b) salinity, (c) light attenuation (turbidity), and (d) UV-light fluorescence. Section is following the main Atlantic Intermediate Water (AIW) inflow from Norske Trough (S8-S10) towards the Nioghalvfjerdsbrae (79NG) terminus (S1), and glacial modified AIW (mAIW) outflow from Nioghalvfjerdsfjorden Bay (S1, S2), Westwind Trough (S3, S5) towards the shelf break (S6) and Fram Strait (S7). The isobar (white contours, in kg/m^3) $\sigma_\Theta = 27.73$ distinguishes between AIW and mAIW. Approximately two thirds of the subglacial cavity outflow is contained between $\sigma_\Theta = 27.5\text{-}27.73\ kg/m^3$ corresponding to depths of ~120-270 m at S1. Polar Surface Water (including glacial runoff and sea-ice melt) is present at $\sigma_\Theta < 26.1\ kg/m^3$. Black dots indicate location of discrete large-CTD measurements; vertical lines (bold black) indicate data obtained from ultraclean CTD measurements. Ultraclean CTD station numbers are indicated in ‘a’.

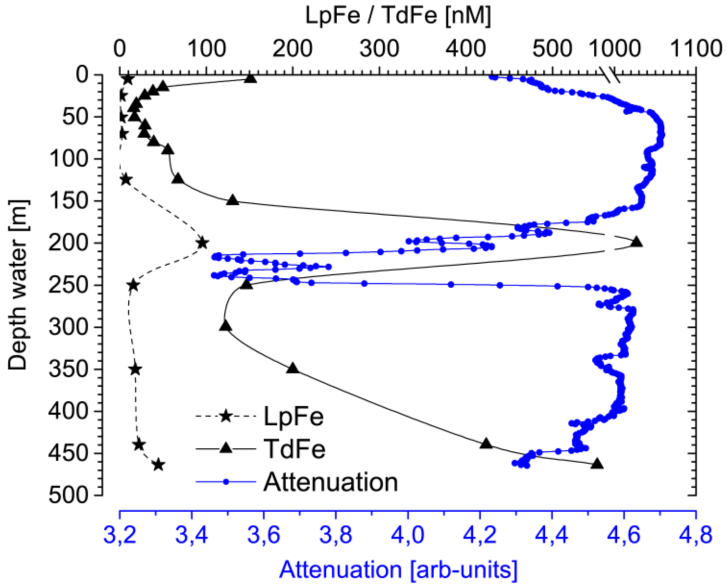

Supplementary Figure 2: Depth profiles of Fe at the Nioghalvfjerdsbrae (79NG) terminus (S1). Labile particulate Fe (LpFe, black stars), total dissolvable Fe (TdFe, black triangles) and light attenuation (i.e. turbidity, blue dots). A correlation between LpFe, TdFe and turbidity is evident, particularly at the modified Atlantic Intermediate Water outflow (~120-270 m depth).

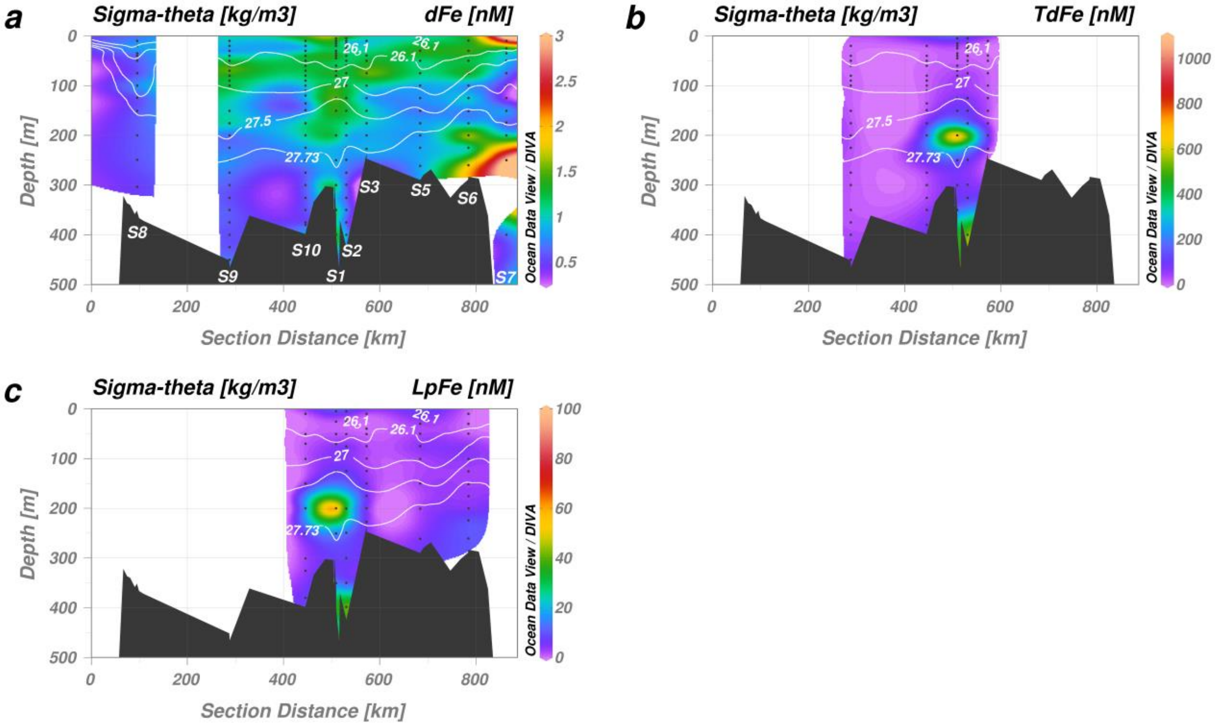

Supplementary Figure 3: NE Greenland Shelf section for Fe passing the Nioghalvfjerdsbrae (79NG) glacier terminus (S1). Isobars (white contours) as per Supplementary Fig. 1. Black dots indicate location of ultraclean CTD sampling. Ultraclean CTD station numbers are indicated in 'a'. (a) Dissolved Fe (dFe), (b) total dissolvable Fe (TdFe), and (c) labile particulate Fe (LpFe) following the main Atlantic Intermediate Water (AIW) inflow towards the glacier terminus (S1) and the main modified AIW outflow towards Fram Strait. Station bottom depth is applied to define a basic bathymetry for clarity.

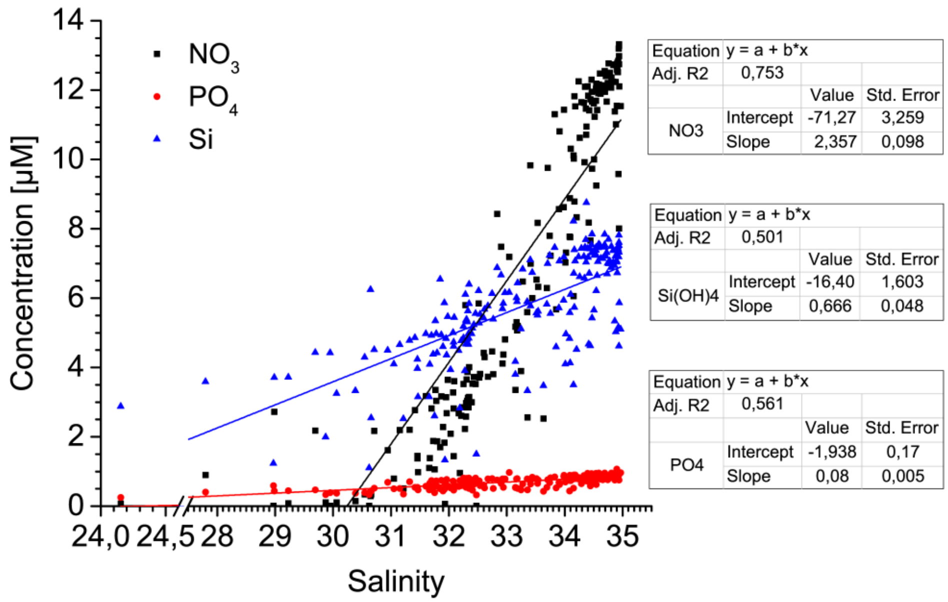

Supplementary Figure 4: Linear regressions for macronutrients across the NE Greenland shelf (S1-6, S8-13). Nitrate (NO_3, black squares), silicic acid ($Si(OH)_4$, blue triangles) and phosphate (PO_4, red dots) concentrations all show a positive relationship with salinity. Error bars not shown for clarity.

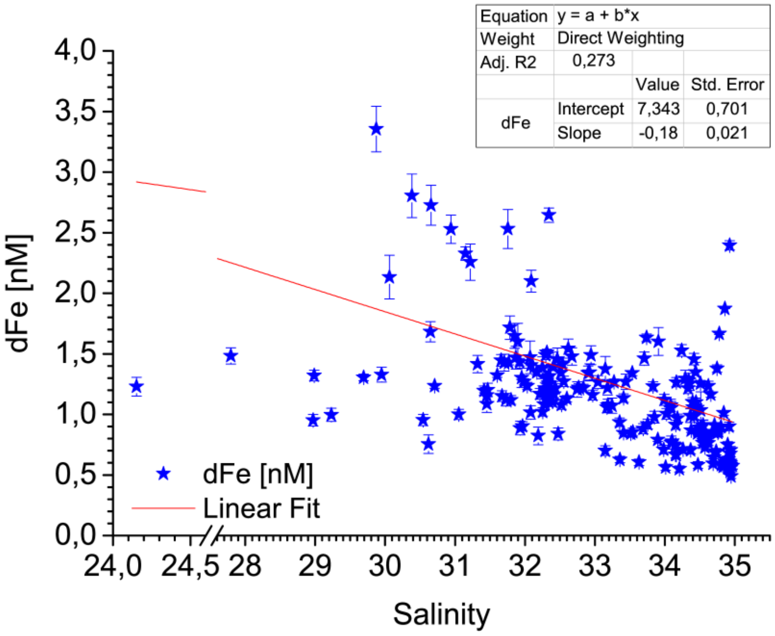

Supplementary Figure 5: Practical salinity versus dissolved Fe (dFe) for all stations on the NE Greenland Shelf: Norske Trough (S8-S10), Nioghalvfjerdsfjorden Bay (S1, S2) including one station near Zachariæ Isstrøm (S11), Dijmphna Sund (S4), Westwind Trough (S3, S5), the shelf break (S6), and two stations on the Central (S12) and outer NE Greenland Shelf (S13). Standard error (blue bars) was included in the linear approximation through program-based direct weighting.

Supplementary Notes

Nutrient fluxes, dilution and sinks across salinity gradients

From a freshwater perspective, a glacier outflow will always result in a positive flux of any chemical component into the marine environment[4]. Yet this can still result in a negative change in the availability of a nutrient in the ocean either by dilution[5,6], as a result of prolific non-conservative removal in estuaries[5], or because the stratification driven by freshwater discharge decreases vertical mixing and thereby suppresses marine primary production[7]. This results in inconsistent terminology between research fields because what always constitutes a nutrient flux at 0 salinity can also be a nutrient sink when considering a flux gate after the major non-conservative mixing processes have occurred or using a box model. Similarly, what always constitutes a nutrient source into the ocean as a whole (especially when considering geological timescales), can still reduce nutrient availability on annual timescales.

This is exemplified by the behavior of phosphate (PO_4). Freshwater PO_4 concentrations are generally low across the cryosphere relative to saline waters[4,5]. Furthermore, PO_4 seems to be subject to a degree of non-conservative removal downstream of glaciers related to high turbidity[8,9]. This results in negative PO_4 anomalies (e.g. Supplementary Figure 4), i.e. the concentration of PO_4 is lower than can be explained by mixing processes. Glacier outflows can therefore both dilute marine PO_4 concentrations[5] and result in local net PO_4 removal[8] despite constituting a measurable PO_4 flux based on freshwater measurements[4].

Estuarine removal

The change in salinity, and other properties such as temperature and turbidity across the salinity gradient, affect chemical components to varying degrees. Conservative components are those that scale linearly with salinity during mixing whereas non-conservative components show pronounced positive or negative deviations from the expected conservative mixing line. Dissolved Fe invariably shows a classic non-conservative estuarine mixing behavior with dFe concentrations always lower than expected from conservative mixing due to prolific removal of dFe onto particles[10].

Whilst most literature concerning estuarine mixing of (micro)nutrients concerns temperate river estuaries, non-conservative losses of dFe are also well documented downstream of glacier outflows[11–14] with 76-99% removed at intermediate salinities. Dissolved silicic acid ($Si(OH)_4$) behavior is more variable; some glacier catchments show close to conservative mixing over the observed salinity gradient[15], and others indicate non-conservative addition at salinities lower than ~10[16,17]. Deriving the net fluxes of dFe and $Si(OH)_4$ arising from glacier outflows is challenging, particularly for large marine-terminating systems, because of the general paucity of data close to glaciers where subglacial discharge first enters the marine environment and the few case studies where extensive data is available spanning the salinity gradient[18]. As elsewhere, in glacier fjords nutrient distributions are also affected by uptake by biota and benthic processes that act to

add/remove nutrients from solution over the same spatial/temporal scale where inorganic processes arising from the change in salinity, turbidity, temperature and pH occur.

The non-conservative aspects of dFe and $Si(OH)_4$ across the salinity gradient account for practically all of the order-of-magnitude variation in flux estimates from Greenland into the ocean, depending on what flux gate window is defined[18]. But variation also arises from a process perspective in how fluxes are scaled. For $Si(OH)_4$, a ten-fold difference in the two available flux estimates from the Greenland Ice Sheet occurs despite using similar freshwater and intermediate salinity $Si(OH)_4$ concentrations[5,17].

Applying linear regression to 79NG

A linear regression of any concentration against salinity should derive the approximate freshwater concentration inclusive of non-conservative effects. However, even in the absence of non-conservative chemical effects, Arctic glacier fjords can exhibit v-shaped nutrient distributions across the salinity gradient because of the multi-dimensional nature of saline water inflow at depth and modified water outflow closer to the surface. In the immediate vicinity of a glacier outflow, a transient increase in all macronutrient concentrations with salinity can therefore be observed followed by a steady decline in surface waters due to biological drawdown. The 0-salinity intercept of a linear regression is therefore sensitive to the range of salinity data selected.

Supplementary Table 3: Linear regression for all nutrient data at trace metal clean stations S1-13 (Supplementary Figure 4 and 5).

**Component**	**Gradient ± SE (R^2)**	**Intercept (± SE)**
NO_3	2.357 ± 0.098 (0.75)	-71.3 ± 3.26
PO_4	0.080 ± 0.005 (0.56)	-1.94 ± 0.17
$Si(OH)_4$	0.666 ± 0.0483 (0.50)	-16.4 ± 1.60
dFe	-0.183 ± 0.021 (0.27)	7.34 ± 0.70

Supplementary Table 4: Linear regression for all nutrient data at trace metal clean stations downstream of Nioghalvfjerdsbrae across the section from S1 to S6.

**Component**	**Gradient ± SE (R^2)**	**Intercept (± SE)**
NO_3	2.11 ± 0.125 (0.759)	-62.4 ± 4.12
PO_4	0.0769 ± 0.0053 (0.70)	-1.88 ± 0.175
$Si(OH)_4$	0.636 ± 0.047 (0.674)	-15.2 ± 1.54
dFe	-0.113 ± 0.024 (0.198)	4.96 ± 0.789

The 0 salinity intercepts for all macronutrients downstream of 79NG are negative. Whilst this suggests a minor role for freshwater outflow on the scale of the region sampled, it is not particularly informative concerning the freshwater concentration as it mainly reflects the v-shaped macronutrient distribution seen in other Greenland fjord systems when considering data across a

salinity gradient from ~10-35[18]. For dFe, conversely, the intercept is positive (7.3 ± 0.7 nM for the region, or 5.0 ± 0.8 nM for stations S1-6) suggesting a freshwater concentration of ~5-7 nM. This is derived from saline data with S > 24, so the intercept is informative concerning the dFe remaining after non-conservative loss and not the freshwater concentration before this loss (which would invariably be much higher)[11,13,14].

General Additive Model (GAM)

GAMs are more useful than linear regressions because they are able to optimize a non-linear fit to multiple parameters simultaneously. Some nutrients clearly display similar spatial trends that can be explained by the same key factors. A Redundancy Analysis clearly shows that nitrate (NO_3), PO_4, and $Si(OH)_4$ cluster together, as do the dissolved metals Fe, Co and Mn (Figure 6). This suggests that similar factors explain their distributions. Relatively good (R^2 >0.75) fits were obtained for all nutrients with a GAM generated by the interaction between salinity and the distance to glacier terminus demonstrating that these variables were able to explain most of the variance in nutrient datasets and these fits were used to obtain estimated 0 salinity concentrations at the glacier terminus (Figure 7).

Supplementary Table 5: GAM-modelled estimates of freshwater concentrations at 0 salinity.

	**GAM fit R^2 (p-value)**	**Estimate 0 salinity**
dFe	0.753 (<0.001)	3.13 ± 0.96 nM
$Si(OH)_4$	0.878 (<0.001)	-12.8 ± 1.96 µM
NO_3	0.965 (<0.001)	-35.5 ± 3.55 µM
PO_4	0.944 (<0.001)	-1.44 ± 0.150 µM

In all cases, a GAM fit was very good and the predicted intercepts were very similar to that predicted from linear regression. In the case of dFe, a positive intercept (3.13 ± 0.96 nM) is consistent with the dFe enrichment expected from fresh water and is, as per linear regression, a value derived after the majority of non-conservative loses have occurred. The negative predictions for macronutrients again verify that there must be a change in gradient for all macronutrients within the subglacial cavity. The differences between linear regression and GAMs in terms of the predicted intercept can be explained by considering that the GAM fit includes variables other than salinity (i.e. distance to glacier terminus), although salinity remains a major factor in explaining the variance in all nutrient datasets (as is shown by RDA).

Contrasting concentrations of macronutrients in mAIW and AIW

To add robustness to our discussion we test how changes in the collection of stations used to assess Atlantic Intermediate Water (AIW) and modified AIW (mAIW) properties would affect our interpretation. For trace metal data, all stations on the shelf are included within the main text. For macronutrients, there is additional data from the large volume CTD that expands the data available.

Here we define mAIW (27.00-27.73 kg/m^3) and AIW (>27.73 kg/m^3) using the same density definition throughout to different subsets of all the large CTD cruise data. The standard deviation of measurements is generally lower using the more extensive combined datasets, although the means remain similar.

From an oceanographic perspective concerning the detection of any changes attributable to local input from the 79NG, the most meaningful definitions of mAIW are those determined immediately adjacent to the ice-tongue as this determines the properties of outflow before any extensive nutrient drawdown, or dilution of mAIW can occur. The most meaningful definition of AIW is that determined at the deepest stations outside the fjord and upstream of the glacier outflow as this precludes any local processes in addition to those occurring underneath the 79NG ice-tongue (e.g. benthic inputs across the shelf)[19] which could affect the properties of AIW as it flows along the fjord prior to entering underneath the subglacial cavity.

Supplementary Table 6: Concentrations of nitrate (NO_3), phosphate (PO_4), silicic acid ($Si(OH)_4$) and dissolved Fe (dFe) in Atlantic Intermediate Water (AIW) and modified AIW following different boundary conditions.

	**Boundaries (clean stations in zone)**	**NO_3 [µM]**	**PO_4 [µM]**	**$Si(OH)_4$ [µM]**	**dFe (refers to S stations only) [nM]**
mAIW	(i) Inner-fjord 79.399-79.724° N, 16.150° W (S1,2,3 and 4)	11.45 ± 1.41	0.796 ± 0.119	7.57 ± 3.20*<br>7.29 ± 0.83	1.18 ± 0.24
mAIW	(ii) Inner-fjord 79.399-79.724° N, 18.680° W, (S1, 2 and 4)	11.55 ± 1.34	0.792 ± 0.123	7.63 ± 3.56*<br>7.30 ± 0.852	1.28 ± 0.22
AIW	(a) S10 and associated cross-section	12.46 ± 0.31	0.964 ± 0.013	7.25 ± 0.18	0.61 ± 0.09
AIW	(b) S2 and associated cross-section	12.44 ± 0.30	0.935 ± 0.007**	7.47 ± 0.087	0.72 ± 0.11
AIW	(c) AIW (combined S10 and S2 sections)	12.45 ± 0.30	0.960 ± 0.016	7.38 ± 0.17	0.67 ± 0.11

*One outlier in the silicic acid dataset has a large effect on the mean with (*), or without, its inclusion. For further data analysis the outlier was not excluded. ** This refers to only two measurements of PO_4 and is thus not considered further.

Comparing the mAIW and AIW concentrations of macronutrients suggests that there is possibly a decline in NO_3 concentration between AIW and mAIW within Nioghalvfjerdsfjorden, but whether or not this is the case is sensitive to the definition of mAIW and only significant ($p < 0.05$) when using the broader definition of mAIW. This suggests that NO_3 loss is not specifically related to processes occurring within the cavity and likely reflects biological drawdown of NO_3 beyond the ice-tongue at stations downstream of S1 (Supplementary Figure 1D). For PO_4, the changes between mAIW and AIW are more evident than for NO_3 as a statistically significant loss ($p < 0.05$) of PO_4 is determined when comparing AIW with mAIW outflow by any definition. This may therefore partially reflect a process occurring under the ice cavity as the difference is already evident at the ice-tongue. Some low PO_4 concentrations are evident at S1 when considering the section S1 to S3 and this is similar to observations in Sermilik Fjord (East Greenland)[8]. For $Si(OH)_4$, there is no significant difference between any of the defined water masses (ANOVA, $p > 0.9$).

Supplementary Table 7: Level of significance (p-value) in glacial nitrate (NO_3) decline following transformation of Atlantic Intermediate Water (AIW) to modified AIW tested by a set of different boundary conditions (Supplementary Table 6).

**NO_3**	**mAIW (i)**	**mAIW (ii)**
AIW (a)	0.039	Not significant
AIW (b)	0.010	Not significant
AIW (c)	<0.001	Not significant

Supplementary Table 8: Level of significance (p-value) for glacial phosphate (PO_4) decline following transformation of Atlantic Intermediate Water (AIW) to modified AIW tested by a set of different boundary conditions (Supplementary Table 6).

**PO_4**	**mAIW (i)**	**mAIW (ii)**
AIW (a)	<0.001	<0.001
AIW (b)	Not tested**	Not tested**
AIW (c)	<0.001	<0.001

Supplementary Table 9: Stable Fe isotope composition (δ^{56}Fe) in dissolved Fe (dFe) samples of the NE Greenland Shelf, the East Greenland Current (EGC), and the West Spitsbergen Current (WSC), expressed relative to IRMM-014. Samples were pooled according to water mass properties. Measurement uncertainties for δ^{56}Fe are reported as two standard error of the mean of analysis (2SE, 2σ; see Methods).

**Station**	**Latitude [°N]**	**Longitude [°E]**	**Depth [m]**	**dFe [nM]**	**δ^{56}Fe [‰]**	**2SE [‰]**	
S1	79.5688	-19.5180	25	2.3	-0.12	0.09	79NG surface discharge (PSW)
			125-200	1.3-1.5	+0.07	0.09	79NG cavity discharge (mAIW)
S11	79.2873	-18.0978	10-19	1.4-2.5	-0.89	0.09	Zachariæ Isstrøm surface discharge
			39	2.6	-0.60	0.09	
	79.0003	7.4983	85-125	0.5-0.7	+0.15	0.09	WSC Atlantic Water[A]
	78.9860	6.9943	101-176				

A: Core Atlantic Water of the West Spitsbergen Current (see ref.[20]).

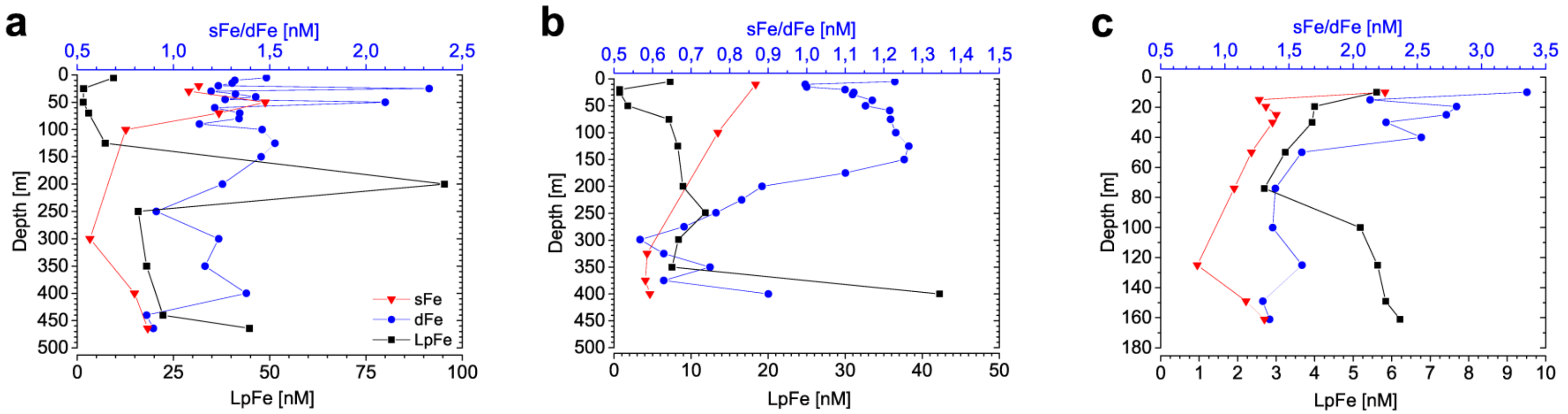

Supplementary Figure 6: Depth profiles of Fe at stations S1, S2 and S4. Soluble Fe (sFe, red triangles), dissolved Fe (dFe, blue dots) and labile particulate Fe (LpFe, black squares) for the (a) Nioghalvfjerdsbrae (79NG) terminus (S1), (b) Nioghalvfjerdsfjorden Bay (S2) and (c) the 79NG side-exit Dijmphna Sund (S4, sill station). Error bars not shown for clarity.

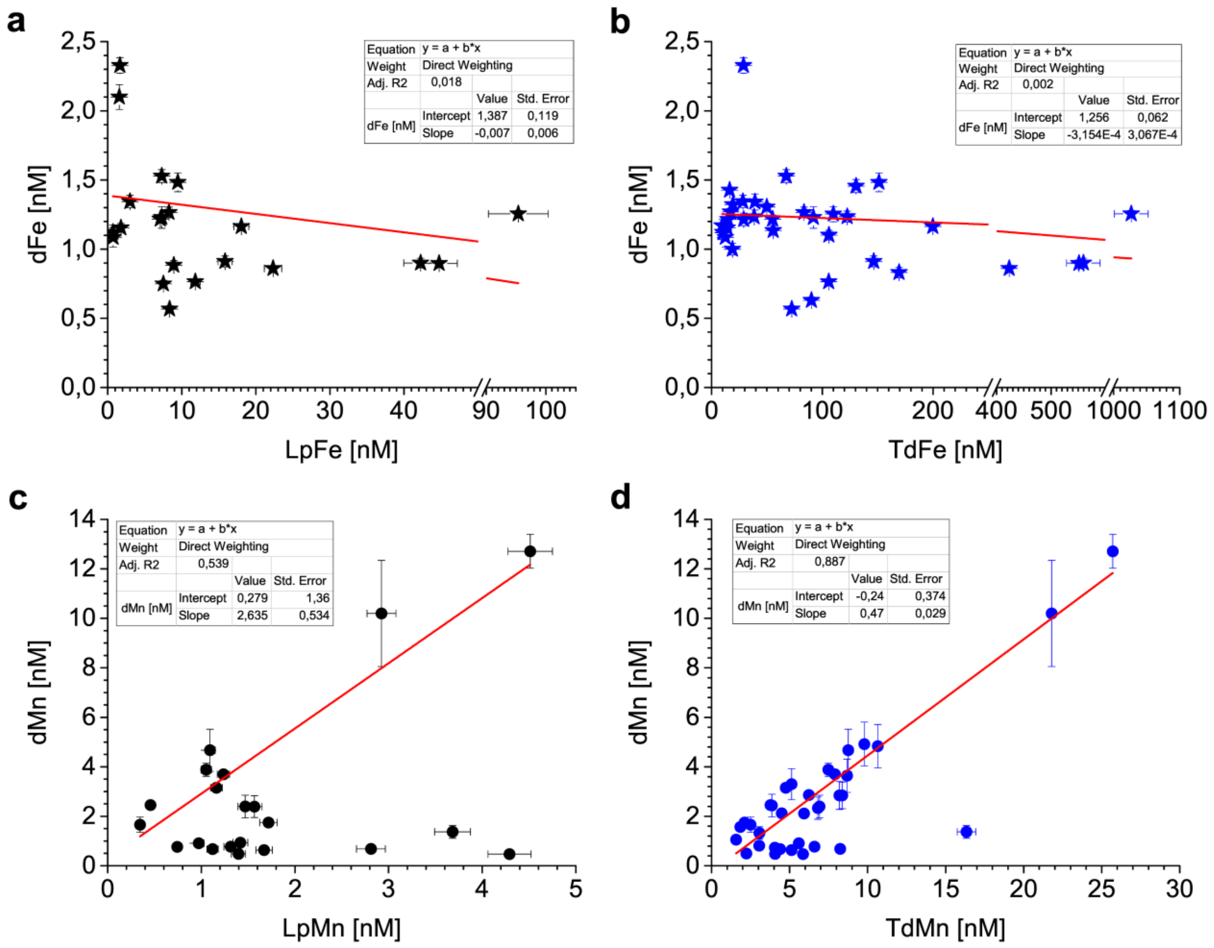

Supplementary Figure 7: A comparison of dissolved versus particulate fractions of Fe and Mn. (a) Dissolved Fe (dFe) and labile particulate Fe (LpFe, black stars), (b) dFe and total dissolvable Fe (TdFe, blue stars), (c) dissolved Mn (dMn) and labile particulate Mn (LpMn, black dots), and (d) dMn and total dissolvable Mn (TdMn, blue dots) for stations S1 and S2 downstream to the Nioghalvfjerdsbrae (79NG) glacier terminus. Standard errors are depicted as whiskers and included in the calculation of linear fits (red line) by direct weighting.

References

1. Bruland Research Lab. Consensus Values for the GEOTRACES 2008 and SAFe Reference Samples. (2009). Available at: https://websites.pmc.ucsc.edu/~kbruland/GeotracesSaFe/kwbGeotracesSaFe.html. (Accessed: 12th March 2019)
2. Rapp, I., Schlosser, C., Rusiecka, D., Gledhill, M. & Achterberg, E. P. Automated preconcentration of Fe, Zn, Cu, Ni, Cd, Pb, Co, and Mn in seawater with analysis using high-resolution sector field inductively-coupled plasma mass spectrometry. *Anal. Chim. Acta* **976**, 1–13 (2017).
3. Joint Research Centre. Certified Reference Material BCR– 414. (2017). Available at: https://crm.jrc.ec.europa.eu/p/q/bcr+414/BCR-414-PLANKTON-trace-elements/BCR-414. (Accessed: 21st January 2020)
4. Wadham, J. L. *et al.* Ice sheets matter for the global carbon cycle. *Nat. Commun.* **10:3567**, (2019).
5. Meire, L. *et al.* High export of dissolved silica from the Greenland Ice Sheet. *Geophys. Res. Lett.* **43**, 9173–9182 (2016).
6. Höfer, J. *et al.* The role of water column stability and wind mixing in the production/export dynamics of two bays in the Western Antarctic Peninsula. *Prog. Oceanogr.* **174**, 105–116 (2019).
7. Holding, J. M. *et al.* Seasonal and spatial patterns of primary production in a high-latitude fjord affected by Greenland Ice Sheet run-off. *Biogeosciences* **16**, 3777–3792 (2019).
8. Cape, M. R., Straneo, F., Beaird, N., Bundy, R. M. & Charette, M. A. Nutrient release to oceans from buoyancy-driven upwelling at Greenland tidewater glaciers. *Nat. Geosci.* **12**, 34–39 (2019).
9. Cantoni, C. *et al.* Glacial Drivers of Marine Biogeochemistry Indicate a Future Shift to More Corrosive Conditions in an Arctic Fjord. *J. Geophys. Res. Biogeosciences* **125**, (2020).
10. Boyle, E. A., Edmond, J. M. & Sholkovitz, E. R. The mechanism of iron removal in estuaries. *Geochim. Cosmochim. Acta* **41**, 1313–1324 (1977).
11. Kanna, N., Sugiyama, S., Fukamachi, Y., Nomura, D. & Nishioka, J. Iron Supply by Subglacial Discharge Into a Fjord Near the Front of a Marine-Terminating Glacier in Northwestern Greenland. *Global Biogeochem. Cycles* **34**, e2020GB006567 (2020).
12. Hopwood, M. J. *et al.* Seasonal changes in Fe along a glaciated Greenlandic fjord. *Front. Earth Sci.* **4:15**, (2016).
13. Zhang, R. *et al.* Transport and reaction of iron and iron stable isotopes in glacial meltwaters on Svalbard near Kongsfjorden: From rivers to estuary to ocean. *Earth Planet. Sci. Lett.* **424**, 201–211 (2015).
14. Schroth, A. W., Crusius, J., Hoyer, I. & Campbell, R. Estuarine removal of glacial iron and implications for iron fluxes to the ocean. *Geophys. Res. Lett.* **41**, 3951–3958 (2014).
15. Brown, M. T., Lippiatt, S. M. & Bruland, K. W. Dissolved aluminum, particulate aluminum, and silicic acid in northern Gulf of Alaska coastal waters: Glacial/riverine inputs and extreme reactivity. *Mar. Chem.* **122**, 160–175 (2010).
16. Kanna, N. *et al.* Upwelling of Macronutrients and Dissolved Inorganic Carbon by a Subglacial Freshwater Driven Plume in Bowdoin Fjord, Northwestern Greenland. *J. Geophys. Res. Biogeosciences* **123**, 1666–1682 (2018).

17. Hawkings, J. R. *et al.* Ice sheets as a missing source of silica to the polar oceans. *Nat. Commun.* **8:14198**, (2017).

18. Hopwood, M. J. *et al.* Review Article: How does glacier discharge affect marine biogeochemistry and primary production in the Arctic? *Cryosph.* **14**, 1347–1383 (2020).

19. Hong, C. N. *et al.* Sediment efflux of silicon on the Greenland margin and implications for the marine silicon cycle. *Earth Planet. Sci. Lett.* **529:115877**, (2020).

20. Krisch, S. *et al.* The influence of Arctic Fe and Atlantic fixed N on summertime primary production in Fram Strait, North Greenland Sea. *Sci. Rep.* **10:15230**, (2020).

Plotting

Supplementary Figures 1 and 3 were made using Ocean Data View, version 5.3.0 (Schlitzer, R., Ocean Data View, https://odv.awi.de, 2020). All other Supplementary Figures were made with Origin(Pro) software, version 9.1.0. (OriginLab Corporation, Northampton, MA, USA). All Supplementary Figures were produced by S.K.
